# Advanced Glycation End Products Increase Salivary Gland Hypofunction in d-Galactose-Induced Aging Rats and Its Prevention by Physical Exercise

**DOI:** 10.3390/cimb43030142

**Published:** 2021-11-19

**Authors:** Woo Kwon Jung, Su-Bin Park, Hyung Rae Kim, Hwa Young Ryu, Yong Hwan Kim, Junghyun Kim

**Affiliations:** Department of Oral Pathology, School of Dentistry, Jeonbuk National University, Jeonju 54896, Korea; wkjungjbnu@gmail.com (W.K.J.); tnqls309@gmail.com (S.-B.P.); rlagudfo31@gmail.com (H.R.K.); naive17jbnu@gmail.com (H.Y.R.); kg7229ku@gmail.com (Y.H.K.)

**Keywords:** advanced glycation end products, physical exercise, salivary gland

## Abstract

A declined salivary gland function is commonly observed in elderly people. Advanced glycation end products (AGEs) are believed to contribute to the pathogenesis of aging. Although physical exercise is shown to increase various organ functions in human and experimental models, it is not known whether it has a similar effect in the salivary glands. In the present study, we evaluated the AGEs burden in the salivary gland in the aging process and the protective effect of physical exercise on age-related salivary hypofunction. To accelerate the aging process, rats were peritoneally injected with D-galactose for 6 weeks. Young control rats and d-galactose-induced aging rats in the old group were not exercised. The rats in the physical exercise group ran on a treadmill (12 m/min, 60 min/day, 3 days/week for 6 weeks). The results showed that the salivary flow rate and total protein levels in the saliva of the d-galactose-induced aging rats were reduced compared to those of the young control rats. Circulating AGEs in serum and secreted AGEs in saliva increased with d-galactose-induced aging. AGEs also accumulated in the salivary glands of these aging rats. The salivary gland of aging rats showed increased reactive oxygen species (ROS) generation, loss of acinar cells, and apoptosis compared to young control mice. However, physical exercise suppressed all of these age-related salivary changes. Overall, physical exercise could provide a beneficial option for age-related salivary hypofunction.

## 1. Introduction

Most elderly people have a clinical symptom of age-related hyposalivation [1]. The maintaining of normal salivary function is important for a healthy oral environment. The hypofunction of the salivary gland can induce several clinical discomforts, such as swallowing problems, xerostomia, and halitosis [2]. Furthermore, this functional disorder of the salivary gland worsens quality of life and increases the prevalence of various oral diseases, such as periodontitis, mucositis, and tooth decay [3]. The pathogenic risk factors of salivary gland hypofunction are chemical medications, several chronic diseases, radiation therapy, and the aging process [4]. To control this clinical symptom, there are several treatment options, such as artificial saliva, chewing gum, malic acid, and pilocarpine [5]. However, these medications only provide temporary relief from symptoms by increasing salivary flow.

Advanced glycation end products (AGEs) are non-enzymatic modifications of proteins or lipids with sugars. AGEs form in vivo in hyperglycemic environments and during aging. [6,7]. AGEs accumulate in various tissues and bind to AGE-specific receptors (RAGEs), which play an important role in the development of age-related organ hypofunction [8]. Evidence shows that AGEs can cause the generation of reactive oxygen species (ROS) [9]. Cells in the human body are chronically exposed to oxidative stress during the aging process, causing cell injury [10]. The irreversible formation of AGEs increases the risk of developing periodontitis and pulptitis in oral tissues [11]. RAGE is present in the salivary gland and is highly expressed in the salivary gland with Sjogren syndrome [12]. In humans and animals, the rate of accumulation of AGE correlates inversely with species longevity [13]. However, it is not clear whether AGE accumulation is due to aging or age-related salivary gland hypofunction.

Regular physical exercise can remove oxidative stress and AGEs burden [14,15]. Moderate exercise induces an enhanced expression of antioxidant enzymes, resulting in a decrease in oxidative stress [16]. Physical exercise decreased the prevalence of periodontal disease [17] and increased the salivary flow rate, protein, and lysozyme secretion [18]. Thus, in the present study, we evaluated the AGEs burden in the aging salivary gland. In addition, we examined the effects of physical exercise on age-related salivary gland hypofunction.

## 2. Results

### 2.1. Physical Exercise Improves Hyposalivation in d-Galactose-Induced Aging Rats

**Figure 1:**
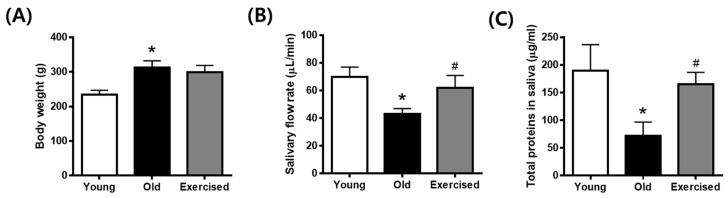
Salivary gland hypofunction in d-galactose-induced aging rats. (**A**) Body weight, (**B**) salivary flow rate, (**C**) total protein levels in saliva. Values in the bar graphs represent the means ± SE, *n* = 8. * *p* < 0.05 vs. young group, # *p* < 0.05 vs. old group.

Body weight in the d-galactose-injected old group increased slightly compared to that in the young control group and was not significantly altered by physical exercise (Figure 1A). The salivary flow rate was reduced in d-galactose-induced aging rats, but reduced saliva secretion was significantly recovered following physical exercise (Figure 1B). Based on saliva analysis, the total secreted proteins were decreased in aging rats and significantly increased in the exercise group (Figure 1C). These data suggest that aging individuals suffer from salivary hypofunction due to decreased saliva secretion, which can be alleviated by physical exercise.

### 2.2. Physical Exercise Decreases the Levels of Circulating AGEs in Serum and Secreted AGEs in Saliva

**Figure 2:**
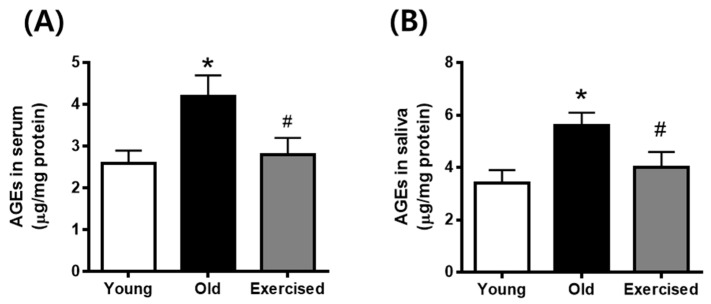
Circulating AGEs levels in serum (**A**) and secreted AGEs levels in saliva (**B**) from d-galactose-induced aging rats. AGE levels were determined by ELISA. Values in the bar graphs represent the means ± SE, *n* = 8. * *p* < 0.05 vs. young group, # *p* < 0.05 vs. old group.

To determine whether physical exercise reduced the AGEs formation in the body, we examined the levels of circulating AGEs in serum and secreted AGEs in saliva. As shown in Figure 2, both circulating and secreted levels of AGEs in aging rats were significantly higher compared to young control rats. Physical exercise decreased the levels of circulating and secreted AGEs compared with the old control group.

### 2.3. Physical Exercise Decreases the Accumulation of AGEs in the Salivary Gland

**Figure 3:**
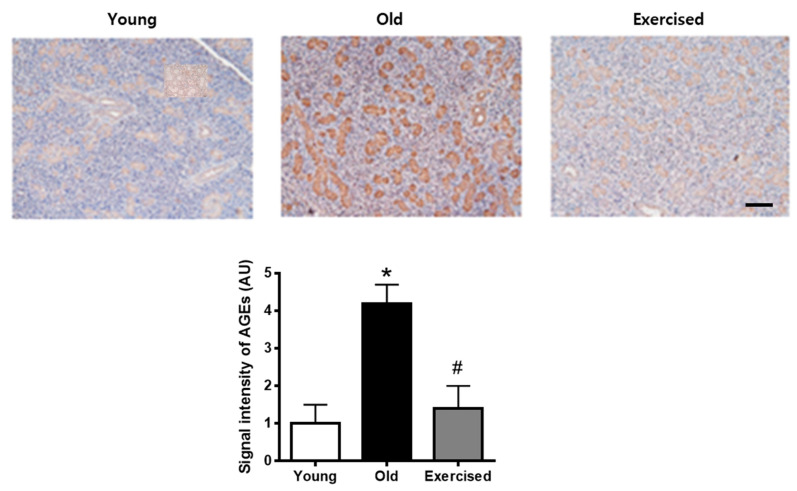
AGE accumulations in the salivary gland of d-galactose-induced aging rats. Immunohistochemical staining for AGEs in the salivary gland. Scale bar indicates 500 μm. Values in the bar graphs represent the means ± SE, *n* = 8. * *p* < 0.05 vs. young group, # *p* < 0.05 vs. old group.

To evaluate the burden of salivary AGEs in d-galactose-induced aging rats, we examined the immunohistochemical staining of AGEs in the salivary gland tissues. As shown in Figure 3, AGEs accumulations were largely increased in both acinar and tubular cells compared with the young control rats. Physical exercise significantly prevented AGEs accumulations in the aging rats to a level similar to that observed in young control rats.

### 2.4. Physical Exercise Decreases the Generation of ROS in the Salivary Gland

**Figure 4:**
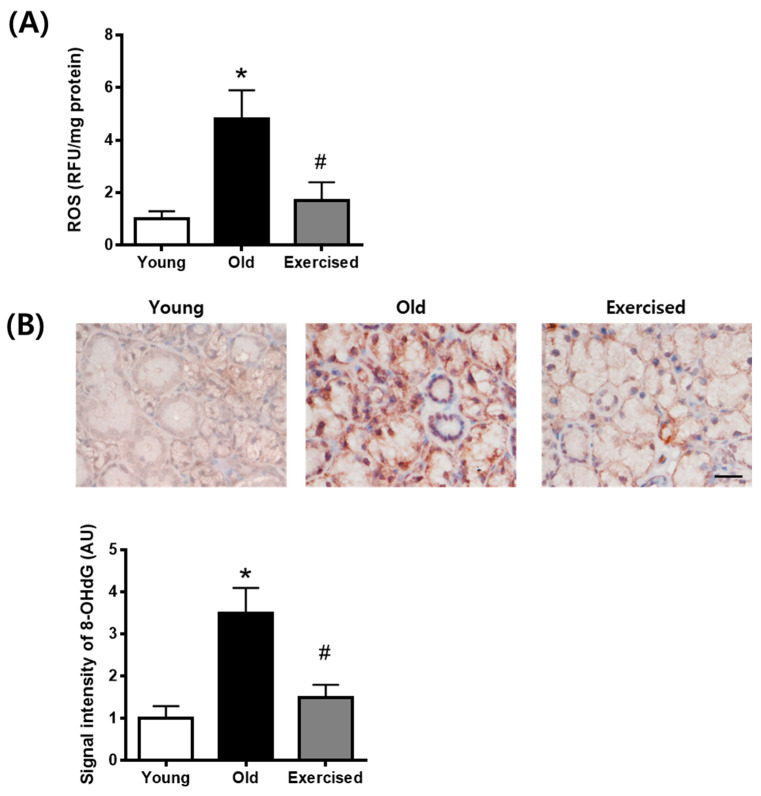
ROS generation in the salivary gland from d-galactose-induced aging rats. (**A**) ELISA assay for ROS in the salivary gland. (**B**) Immunohistochemical staining for 8-OHdG in the salivary gland. Scale bar indicates 200 μm. Values in the bar graphs represent the means ± SE, *n* = 8. * *p* < 0.05 vs. young group, # *p* < 0.05 vs. old group.

To evaluate salivary ROS levels in d-galactose-induced aging rats, we examined ROS ELISA assay and immunohistochemical staining of 8-hydroxy-2-deoxyguanosine (8-OHdG), an oxidative DNA damage marker, in salivary gland tissues. As shown in Figure 4, ROS generation and 8-OHdG expression were largely increased in the d-galactose-induced aging rats. Physical exercise significantly prevented the generation of ROS and oxidative DNA damage in the aging rats.

### 2.5. Physical Exercise Inhibits Apoptosis in the Salivary Glands of d-Galactose-Induced Aging Rats

**Figure 5:**
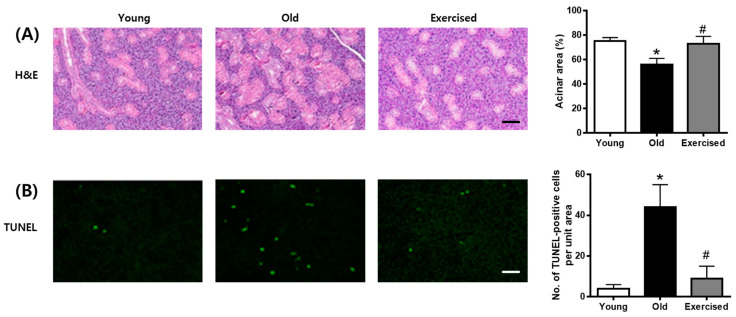
Histopathological changes and apoptosis in the salivary gland from d-galactose-induced aging rats. (**A**) Representative salivary glands were stained with H&E. (**B**) Representative TUNEL staining in the salivary glands. Scale bar indicates 500 μm. TUNEL-positive cells were determined. Values in the bar graphs represent the means ± SE, *n* = 8. * *p* < 0.05 vs. young group, # *p* < 0.05 vs. old group.

H&E staining revealed that the number of acinar cells decreased, but the number of convoluted tubes was increased in d-galactose-induced aging rats. These histopathological changes in the aging rats were ameliorated by physical exercise (Figure 5A). TUNEL staining was used to determine whether acinar cells die due to apoptosis. As shown in Figure 4B, apoptosis was detected by TUNEL staining and was increased in the old group and restored by physical exercise (Figure 5B). Physical exercise protects salivary gland tissues by reducing apoptosis.

## 3. Discussion

The gradual decline in salivary function is commonly observed in older people. The patients with salivary hypofunction have a high risk factor for developing oral diseases such as tooth decay and periodontitis [19,20]. Thus, the age-related decline in salivary function should be managed to maintain the healthy oral environment. In this study, we assessed physical exercise to improve salivary gland function by reducing the burden of AGEs.

Our study showed evidence that an increased burden of AGEs occurred in the salivary gland of aging rats. The functional alteration of the salivary gland was significantly induced in aging rats compared to young control rats. These findings indicate that the significant burden of AGEs in the salivary glands led to the significant hypofunction of this tissue. This hyposalivation is in agreement with the results of Yamauchi et al. [21], who observed a decrease in salivary flow rate and increased oxidative stress in 72-week-old mice. Our results described the link between age-related hypofunction of the salivary gland and AGEs burden. In addition, we showed that physical exercise exerts preventive effects on salivary glands in aging rats.

Zhang et al. reported that the injection of a low dose of d-galactose into mice could induce changes that resembled accelerated aging [22]. This d-galacotose-induced aging process included a decreased neuromuscular activity, increased production of free radicals, decreased anti-oxidant enzyme activity, and diminished immune response [23]. Because these biochemical and physiological changes resemble observations in the normal aging process, the d-galactose-induced aging model in mouse and rat is widely used for aging research and drug testing [24]. The underlining mechanism, responsible for d-galactose-induced aging changes, remains largely unknown.

d-galactose is a reducing sugar that reacts easily with free amines of amino acids in proteins and peptides, both in vitro and in vivo, to form AGEs [25]. The hypothesis here is that accumulated d-galactose may react with proteins and peptides to form AGEs in vivo and that the increased AGEs can accelerate the aging process. Song et al. showed that d-galactose-injected mice had a significant increase in serum AGE levels, similar to aged controls [26]. Both d-galactose- and exogenous AGE-treated mice, resembling aged mice, suggest that AGEs, at least partially, account for the mechanism of this aging model. Furthermore, aminoguanidine, a well-known AGE inhibitor, prevented d-galactose-induced aging changes. These results suggest that glycation, rather than free radicals, is the main cause of aging in this animal model.

The cytotoxic effects of AGEs were shown in several studies [27]. Since the body does not contain any enzymes capable of structural degradation of AGEs, AGEs can accumulate in many tissues [28]. The interaction between AGE and its receptor can induce ROS overproduction [29]. Oxidative stress is an important feature of aging [30]. ROS production was accelerated under disease conditions and during the aging process [10], and frequently contributed to the tissue-damaging effects [9,31]. Collectively, these results suggest that the aging process by d-galactose can induce salivary injury through AGEs-related oxidative stress and inflammation, which can lead to salivary gland dysfunction.

Here, we hypothesize that the reduction in AGEs burden by physical exercise may contribute to the inhibition of salivary gland dysfunction. The current study clearly demonstrated that physical exercise restored circulating and secreted AGEs levels to near-normal levels in aging rats, in parallel to a marked increase in salivary flow rate. These findings provide evidence that physical exercise has a beneficial effect on age-related salivary gland hypofunction. Physical exercise also has a positive influence on oxidative status [32,33]. Our previous study showed that physical exercise inhibited the AGEs burden in renal and retinal tissues [34,35,36]. In addition, exercise-induced increased energy demands might decrease the pool of reactive intermediates for glycoxidation or lipoxidation [37].

Acinal cell loss by apoptosis can inevitably affect the salivary flow rate, resulting in salivary hypofunction [38]. Furthermore, high concentrations of AGEs contribute to apoptotic cell death whenever they are generated in the context of the apoptotic process [39,40]. The present study showed that the d-galactose-induced aging process increased the number of TUNEL-positive cells in the salivary gland. However, we found that physical exercise markedly decreased the number of TUNEL-positive cells in aging rats. In previous reports, physical exercise attenuated neuronal cell apoptosis in a rat model of transient middle cerebral artery occlusion [41], and decreased mitochondrial-mediated apoptotic signaling pathways in the aging heart [42]. Therefore, our findings suggest that physical exercise has a potential anti-apoptotic effect in the salivary gland.

In conclusion, our study demonstrates that the AGEs burden was increased in the salivary gland of d-galactose-induced aging rats. The physical exercise has protective effects on the salivary gland of aging rats. These novel findings provide insight into the effects of regular physical exercise against age-related salivary hypofunction.

## 4. Materials and Methods

### 4.1. Animals and Experimental Design

Eighteen male 6-week-old Sprague Dawley rats were randomly divided into 3 groups: young control rats (Con, *n* = 8), d-galactose-induced aging rats (Old, *n* = 8), and d-galactose-induced aging rats with physical exercise (Exercise, *n* = 8). To accelerate aging, d-galactose (100 mg/kg/day) was intraperitoneally injected in the rats in the old and exercise groups for 6 weeks. The rats in the exercise group were forced to run on a motorized treadmill once a day, three times a week for 6 weeks. The treadmill velocity was at a speed of 12 m/min for 60 min. A pace of 12 m/min is considered to be a moderate walk–jog pace for laboratory animals [43]. All of the experimental procedures were performed under the supervision of our Institutional Animal Care and Use Committee (IACUC No. 2018-067). To measure salivary flow rate analysis, rats were injected intraperitoneally with pilocarpine hydrochloride (2 mg/kg, Sigma-Aldrich, St. Louis, MO, USA). Saliva samples were then collected for 15 min. At necropsy, blood samples were collected and the submandibular salivary glands isolated.

### 4.2. Quantification of AGEs in Saliva and Blood

Total protein levels in saliva and serum were examined using a Quick Start™ Bradford protein assay kit (Bio-Rad, Hercules, CA, USA). The AGEs levels were detected using a rat advanced glycation end products ELISA kit (MyBioSource, San Diego, CA, USA) according to the manufacturer’s instructions.

### 4.3. Oxidative Stress Assay in Salivary Gland

Frozen salivary gland tissues were homogenized in lysis buffer (150 mM NaCl, 1% Triton X-100 and 10 mM Tris, pH 7.4) containing protease inhibitor. The homogenate was centrifuged at 10,000× *g* for 10 min at 4 °C and the supernatant was collected for measurement of ROS levels. ROS levels were examined using a Rat Reactive Oxygen Species ELISA Kit (MyBioSource, San Diego, CA, USA) according to the manufacturer’s instructions.

### 4.4. Histopathological Analysis

The salivary gland tissue sections were stained with hematoxylin and eosin (H&E) and examined under light microscopy (BX51, Olympus, Tokyo, Japan).

### 4.5. Apoptosis Analysis

Apoptosis was determined using an in situ cell death detection kit (Roche, Mannheim, Germany) according to the manufacturer’s instructions. The numbers of TUNEL-positive cells were counted under a fluorescence microscope (BX51, Olympus, Tokyo, Japan).

### 4.6. Immunohistochemistry

Immunohistochemistry was performed as previously described [20]. The primary antibody was mouse anti-AGEs (6D12, TransGenic, Kobe, Japan) and mouse anti-8-OHdG (Abcam, Cambridge, MA, USA). Sections were incubated with the VECTASTAIN ABC kit (Vector Laboratory, California, CA, USA) and visualized with 3,3′-diaminobenzidine tetrahydrochloride. The intensity of immunohistochemical staining was analyzed using image analysis software (ImageJ, NIH, Maryland, MD, USA).

### 4.7. Statistical Analysis

The results were evaluated statistically using a one-way analysis of variance followed by Tukey’s multiple comparison test using GraphPad Prism 6.0 (GraphPad Software, San Diego, CA, USA).

## Data Availability

The data presented in this study are available on request from the corresponding author.

## References

[1] López-Pintor R.M., Casañas E., González-Serrano J., Serrano J., Ramírez L., de Arriba L., Hernández G. (2016). Xerostomia, hyposalivation, and salivary flow in diabetes patients. J. Diabetes Res..

[2] Rao P.K.J., Chatra L., Shenai P., Veena K., Prabhu R.V., Kushraj T., Shetty P., Hameed S. (2014). Xerostomia: Few dry facts about dry mouth. Arch. Med. Health Sci..

[3] Mason A.L., Xu L., Guo L., Garry R.F. (1999). Retroviruses in autoimmune liver disease: Genetic or environmental agents?. Arch. Immunol. Et Ther. Exp..

[4] Saleh J., Figueiredo M.A.Z., Cherubini K., Salum F.G. (2015). Salivary hypofunction: An update on aetiology, diagnosis and therapeutics. Arch. Oral Biol..

[5] Visvanathan V., Nix P. (2010). Managing the patient presenting with xerostomia: A review. Int. J. Clin. Pract..

[6] Goh S.Y., Cooper M.E. (2008). Clinical review: The role of advanced glycation end products in progression and complications of diabetes. J. Clin. Endocrinol. Metab..

[7] Tessier F.J. (2010). The Maillard reaction in the human body. The main discoveries and factors that affect glycation. Pathol. Biol..

[8] Brownlee M., Cerami A., Vlassara H. (1988). Advanced glycosylation end products in tissue and the biochemical basis of diabetic complications. N. Engl. J. Med..

[9] Lu J., Wu D.M., Zheng Y.L., Hu B., Zhang Z.F., Ye Q., Liu C.M., Shan Q., Wang Y.J. (2010). Ursolic acid attenuates D-galactose-induced inflammatory response in mouse prefrontal cortex through inhibiting AGEs/RAGE/NF-kappaB pathway activation. Cereb. Cortex.

[10] Xu H., Chen M., Forrester J.V. (2009). Para-inflammation in the aging retina. Prog. Retin. Eye Res..

[11] Ilea A., Babtan A.M., Bosca B.A., Crisan M., Petrescu N.B., Collino M., Sainz R.M., Gerlach J.Q., Campian R.S. (2018). Advanced glycation end products (AGEs) in oral pathology. Arch. Oral Biol..

[12] Katz J., Stavropoulos F., Bhattacharyya I., Stewart C., Perez F.M., Caudle R.M. (2004). Receptor of advanced glycation end product (RAGE) expression in the minor salivary glands of patients with Sjogren’s syndrome: A preliminary study. Scand. J. Rheumatol..

[13] Sell D.R., Lane M.A., Johnson W.A., Masoro E.J., Mock O.B., Reiser K.M., Fogarty J.F., Cutler R.G., Ingram D.K., Roth G.S. (1996). Longevity and the genetic determination of collagen glycoxidation kinetics in mammalian senescence. Proc. Natl. Acad. Sci. USA.

[14] Asghar M., George L., Lokhandwala M.F. (2007). Exercise decreases oxidative stress and inflammation and restores renal dopamine D1 receptor function in old rats. Am. J. Physiol. Ren. Physiol..

[15] Navarro A., Gomez C., Lopez-Cepero J.M., Boveris A. (2004). Beneficial effects of moderate exercise on mice aging: Survival, behavior, oxidative stress, and mitochondrial electron transfer. Am. J. Physiol. Regul. Integr. Comp. Physiol..

[16] Gomez-Cabrera M.C., Domenech E., Vina J. (2008). Moderate exercise is an antioxidant: Upregulation of antioxidant genes by training. Free Radic. Biol. Med..

[17] Ferreira R.O., Correa M.G., Magno M.B., Almeida A., Fagundes N.C.F., Rosing C.K., Maia L.C., Lima R.R. (2019). Physical activity reduces the prevalence of periodontal disease: Systematic review and meta-analysis. Front. Physiol..

[18] Ligtenberg A.J., Brand H.S., van den Keijbus P.A., Veerman E.C. (2015). The effect of physical exercise on salivary secretion of MUC5B, amylase and lysozyme. Arch. Oral. Biol..

[19] Mealey B.L., Ocampo G.L. (2007). Diabetes mellitus and periodontal disease. Periodontology 2000.

[20] Tsai C., Hayes C., Taylor G.W. (2002). Glycemic control of type 2 diabetes and severe periodontal disease in the US adult population. Community Dent. Oral Epidemiol..

[21] Yamauchi Y., Matsuno T., Omata K., Satoh T. (2017). Relationship between hyposalivation and oxidative stress in aging mice. J. Clin. Biochem. Nutr..

[22] Zhang X., Li W.B., Zhang B.L. (1990). Biochemical changes in D-galactose induced subacute toxicity and mimetic aging in mice. Chinese J Pharm Toxicol.

[23] Gong G.Q., Xu F.B. (1991). Study of aging model in mice. J. China Pharm. Univ..

[24] Li W.B., Wei F., Fan M., Zhang J.L., Zhang B.L., Ma X.C., Yang W.P., Wei W. (1995). Mimetic brain aging effect induced by D-galactose in mice. Chin. J. Pharm. Toxicol..

[25] Vlassara H., Bucala R., Striker L. (1994). Pathogenic effects of advanced glycosylation: Biochemical, biologic, and clinical implications for diabetes and aging. Lab. Invest..

[26] Song X., Bao M., Li D., Li Y.M. (1999). Advanced glycation in D-galactose induced mouse aging model. Mech. Ageing Dev..

[27] Brownlee M. (1995). Advanced protein glycosylation in diabetes and aging. Annu. Rev. Med..

[28] Pokupec R., Kalauz M., Turk N., Turk Z. (2003). Advanced glycation endproducts in human diabetic and non-diabetic cataractous lenses. Graefes Arch. Clin. Exp. Ophthalmol..

[29] Yamamoto Y., Yamamoto H. (2012). Interaction of receptor for advanced glycation end products with advanced oxidation protein products induces podocyte injury. Kidney Int..

[30] Hiona A., Leeuwenburgh C. (2008). The role of mitochondrial DNA mutations in aging and sarcopenia: Implications for the mitochondrial vicious cycle theory of aging. Exp. Gerontol..

[31] Cuzzocrea S., McDonald M.C., Filipe H.M., Costantino G., Mazzon E., Santagati S., Caputi A.P., Thiemermann C. (2000). Effects of tempol, a membrane-permeable radical scavenger, in a rodent model of carrageenan-induced pleurisy. Eur. J. Pharm..

[32] Pechter U., Maaroos J., Mesikepp S., Veraksits A., Ots M. (2003). Regular low-intensity aquatic exercise improves cardio-respiratory functional capacity and reduces proteinuria in chronic renal failure patients. Nephrol. Dial. Transpl..

[33] Moinuddin I., Leehey D.J. (2008). A comparison of aerobic exercise and resistance training in patients with and without chronic kidney disease. Adv. Chronic Kidney Dis..

[34] Kim C.S., Park S., Kim J. (2017). The role of glycation in the pathogenesis of aging and its prevention through herbal products and physical exercise. J. Exerc. Nutr. Biochem..

[35] Kim C.S., Park S., Chun Y., Song W., Kim H.J., Kim J. (2015). Treadmill exercise attenuates retinal oxidative stress in naturally-aged mice: An immunohistochemical study. Int. J. Mol. Sci..

[36] Park S., Kim C.S., Lee J., Suk Kim J., Kim J. (2013). Effect of regular exercise on the histochemical changes of d-galactose-induced oxidative renal injury in high-fat diet-fed rats. Acta Histochem. Cytochem..

[37] Boor P., Celec P., Behuliak M., Grancic P., Kebis A., Kukan M., Pronayova N., Liptaj T., Ostendorf T., Sebekova K. (2009). Regular moderate exercise reduces advanced glycation and ameliorates early diabetic nephropathy in obese Zucker rats. Metabolism.

[38] Stephens L.C., Schultheiss T.E., Price R.E., Ang K.K., Peters L.J. (1991). Radiation apoptosis of serous acinar cells of salivary and lacrimal glands. Cancer.

[39] Mahali S., Raviprakash N., Raghavendra P.B., Manna S.K. (2011). Advanced glycation end products (AGEs) induce apoptosis via a novel pathway: Involvement of Ca^2+^ mediated by interleukin-8 protein. J. Biol. Chem..

[40] Li D.X., Deng T.Z., Lv J., Ke J. (2014). Advanced glycation end products (AGEs) and their receptor (RAGE) induce apoptosis of periodontal ligament fibroblasts. Braz. J. Med. Biol. Res..

[41] Zhang L., Hu X., Luo J., Li L., Chen X., Huang R., Pei Z. (2013). Physical exercise improves functional recovery through mitigation of autophagy, attenuation of apoptosis and enhancement of neurogenesis after MCAO in rats. BMC Neurosci..

[42] Kwak H.B. (2013). Effects of aging and exercise training on apoptosis in the heart. J. Exerc. Rehabil..

[43] Billat V.L., Mouisel E., Roblot N., Melki J. (2005). Inter- and intrastrain variation in mouse critical running speed. J. Appl. Physiol..

